# Transabdominal cerclage during pregnancy: A retrospective single operator series over a quarter century

**DOI:** 10.1002/ijgo.14426

**Published:** 2022-09-19

**Authors:** David R. Hall, Mari van de Vyver

**Affiliations:** ^1^ Department of Obstetrics and Gynaecology Stellenbosch University and Tygerberg Hospital Cape Town South Africa; ^2^ Division of Clinical Pharmacology, Department of Medicine, Faculty of Medicine & Health Sciences Stellenbosch University Cape Town South Africa

**Keywords:** cervical insufficiency, cervix, transabdominal cerclage

## Abstract

**Objective:**

To describe the pregnancy outcomes and complications observed in a series of cases of transabdominal cerclage (TAC), which is reserved for highly selected women with recurrent mid‐trimester pregnancy loss, due to cervical insufficiency.

**Methods:**

A retrospective audit covering 25 years (January 1, 1997 to December 31, 2021) was performed at the Obstetric Special Care division, Tygerberg Academic Hospital in Cape Town, South Africa. All 118 pregnancies from 94 procedures, operated and managed by the principal author were included for descriptive analysis.

**Results:**

Eighty‐four (91.3%) of the 92 first pregnancies after first insertion had successful outcomes. All second and third pregnancies (24/24; 100%) were successful. Eight pregnancies did not achieve viability, two women (2/8) did however achieve a successful pregnancy after a subsequent repeat TAC procedure. For the viable pregnancies (110/118), the median gestational age at delivery was 37 weeks (range 28–39 weeks). The median intraoperative blood loss during cerclage insertion was 100 ml (range 25–750 ml).

**Conclusion:**

In experienced hands, TAC during pregnancy is a safe and effective operation, when other less invasive procedures have failed.

## Synopsis

Transabdominal cerclage during pregnancy is a safe and effective operation, when other less invasive procedures have failed.

## 1 INTRODUCTION

Spontaneous preterm delivery before the third trimester of pregnancy is a difficult challenge. A subset of patients present with cervical insufficiency, representing largely asymptomatic activation and dilatation of the cervix. There is a high likelihood of recurrence in a subsequent pregnancy,^1^ so prophylactic vaginal progesterone or prophylactic cervical cerclage are offered to women with one such previous loss and a cervical length less than 25 mm.^2^ When two or more mid‐trimester losses have occurred, intervention is offered regardless of the cervical length. There is however, considerable variation within guidelines^3^ and vaginal cerclage and progesterone have been used in combination.^4^

When transvaginal cerclage has failed, or is impossible because of a very short or damaged cervix, a transabdominal cerclage (TAC) is considered because it provides physical support at the cervico‐isthmic junction.^5^ It is performed via laparotomy or laparoscopy in early pregnancy, or before conception and requires cesarean delivery, but can be retained for subsequent pregnancies. In a rare multicenter, randomized trial comparing transabdominal and transvaginal cerclage in women with a history of failed cerclage, Shennan et al.^6^ found TAC to be superior. They subsequently commented that although the success rates of laparotomy and laparoscopy cases are equivalent, the evidence base of benefit currently sits with the laparotomy route.^7^ The estimated success rate is more than 80% in typical overviews,^8^ but reported case series are mostly small. During pregnancy the procedure is performed from 11 to 14 weeks,^9^ but is more difficult because of the increased vascularity of the pelvic anatomy and the presence of the fetus. Therefore, some surgeons perform preconception procedures (despite the challenge of subsequent first‐trimester miscarriage where the cerclage may impede the expulsion of a non‐viable pregnancy), to limit the intraoperative and postoperative complications during pregnancy.^10^ In a study spanning 22 years, including 62 preconception and 59 first‐trimester TACs, 50% of the women whose procedure was performed in pregnancy experienced hemorrhage of more than 500 ml, whereas no surgical complications occurred in the preconception group.^10^ Local experience did not confirm the heavy surgical blood loss, but, this important finding required formal documentation.

Once the desperate situation of recurrent mid‐trimester losses with failure of less invasive options is reached, TAC is the only option for women wanting to carry their own pregnancy. However, even in large referral centers, the availability of surgeons sufficiently experienced to safely offer TAC has been extremely limited. This has also been the case in South Africa. In addition, many women requiring the procedure do not present before conception, when the operation is easier. Preconception cerclage lends itself to the laparoscopic approach.^11^

Tygerberg Hospital, a tertiary referral unit, has offered TAC via laparotomy during pregnancy from 1997 through a single, principal surgeon. The obligatory internal audit has carefully tracked the pregnancy success rates and complications, but the series required formal documentation. Internationally, pregnancy outcomes and complications^11^ have been described in a few small and moderately sized studies from high‐income countries,^6^, ^10^, ^11^, ^12^ and collaborations are reflected in systematic reviews and meta‐analyses.^13^, ^14^ However, detailed evidence exclusively from low‐ to middle‐income countries (LMICs) is needed.

This study aimed to carefully describe the pregnancy outcomes and complications of women who underwent TAC at Tygerberg Hospital.

## 2 MATERIALS AND METHODS

A retrospective audit was performed of all women (*n* = 92) who underwent a TAC procedure, where the principal investigator was either the surgeon or first assistant, from January 1, 1997 until December 31, 2021. The location was Tygerberg Academic Hospital, a tertiary referral center in Cape Town, South Africa. All women (*n* = 92) were diagnosed as having cervical insufficiency, at least one failed elective transvaginal cerclage, or a very short (<1 cm) or damaged vaginal portion of the cervix. They all received counseling on risks and benefits, with the aim of performing the open laparotomy procedure between 12 and 14 weeks of pregnancy, or earlier if the nuchal translucency and anomaly scan was already performed. During vaginal speculum examination, vaginitis was excluded clinically, but patients still received oral erythromycin or azithromycin in the week before surgery. General anesthesia was standard practice.

The surgical technique is similar to that described by Sumners et al.^15^ In summary, a Pfannenstiel abdominal incision was preferred, but for obese women a midline vertical incision was performed. After opening the uterovesical peritoneum transversely (about 4 cm), the uterus was slowly lifted out of the abdomen, with cupped hands and fingers closing around the cervix. Following inspection of the paracervical vasculature (anterior and posterior), the thumb (anterior) and middle finger (posterior) of the non‐dominant hand were used to draw the uterine artery laterally. With the posterior finger as a guide, an avascular window was located at the level of the internal cervical os, between the cervix and the ascending branches of the uterine arteries. With a blunt‐tipped needle, a 5‐mm wide, woven permanent tape was drawn through each side, taking care not to damage local veins, and knotted posteriorly after correct tensioning. Bleeding from paracervical veins was dealt with by compression or fine dissection and ligation. Hemostasis was re‐checked after gentle return of the uterus to the abdomen. After abdominal closure, the perineum was inspected for blood or amniotic fluid and a single indomethacin suppository (100 mg) was inserted rectally. The surgeon and anesthetist paid particular, prospective attention to the estimated surgical blood loss, but swabs were not weighed.

Fetal cardiac activity was confirmed before discharge, and standard postoperative care was applied. Late complications included rupture of membranes, repeated second‐trimester loss and intrauterine death. During pregnancy, there was heightened sensitivity to preterm labor with the inherent risk of stitch erosion through contractions, but tocolysis was only administered for standard obstetric indications. Elective cesarean delivery was planned at 37–38 weeks of pregnancy. Successful pregnancies reached viability, defined as 27 weeks of gestation and 800 g fetal weight according to local criteria.

The primary aim of this case series was to determine the pregnancy outcomes, chiefly in terms of the success rate. The secondary aims were to describe the patient profile, and complications. Unsuccessful pregnancies were carefully studied.

Names and hospital numbers of the study population, were known to the principal investigator and held in the clinic records. The principal investigator collected all relevant data from the hospital's electronic health records. This was entered onto an anonymized Microsoft excel spreadsheet for statistical analysis, while a separate log linking case numbers to patient identifiers was kept by the principal investigator in a secure location. Distribution of data was assessed using the Shapiro–Wilk and Kolmogorov–Smirnov tests. Results are reported as number (%) and categorical comparisons using the χ^2^ test. Special investigations are reported using mean ± standard deviation and/or median (range) according to the distribution. Continuous variables were compared using either the *t* test (for normally distributed variables) or Mann–Whitney *U* test (for non‐normally distributed variables). Where significance was sought, a *P* value <0.05 was regarded as significant. Statistical analysis was performed using graphpad prism (8.2.0) (GraphPad). The study was approved by the Human Research and Ethics Committee of Stellenbosch University (N21/09/094), which granted a waiver of consent because of the retrospective audit design.

## 3 RESULTS

**FIGURE 1:**
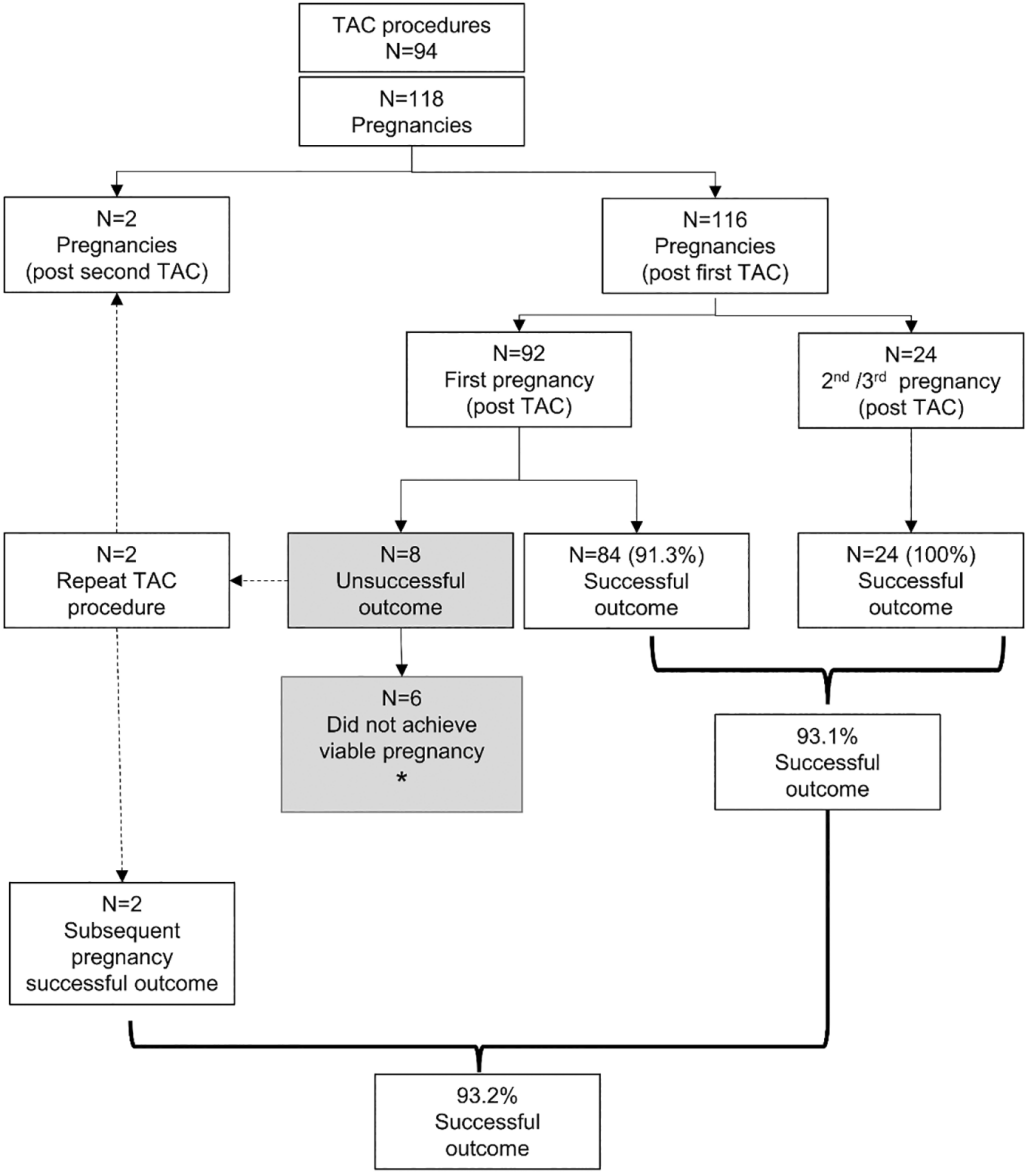
Overview of transabdominal cerclage (TAC) procedures and outcomes. *Two of the six women, who did not achieve a viable pregnancy post TAC, reached viability in a subsequent pregnancy despite removal of TAC, one with a subsequent vaginal cerclage and one unassisted reached 28 weeks of gestation

A total of 118 pregnancies from 94 procedures (92 primary TAC and two repeat TAC), operated and managed by the principal author were included for analysis. A complete data set was available for all cases, with no patients excluded from analysis. Before TAC, patients collectively had 89 first‐trimester miscarriages, 292 second‐trimester miscarriages, and 86 viable pregnancies from 467 pregnancy attempts (success rate 18.4%). Of 92 consecutive (first TAC) cases, the pregnancy during which the TAC was inserted was successful in 84 (91.3%). In two women where the first TAC was unsuccessful and removed, a second TAC was inserted in a subsequent pregnancy, with both achieving success. There were also 24 (22 second, and 2 third) additional pregnancies among 22 women who retained the TAC after the first pregnancy. All were successful bringing the overall success rate to 93.2% (110/118). Six women did not achieve a viable pregnancy after any TAC. Two merit special mention as they achieved viability after TAC removal (one with a repeat vaginal cerclage, the other unassisted, with spontaneous preterm birth at 28 weeks of pregnancy). Only four women in the cohort did not achieve any viable pregnancy. Figure 1 depicts all outcomes. For the 110 viable pregnancies, the median (range) gestational age at delivery was 37 weeks (28–39 weeks).

**FIGURE 2:**
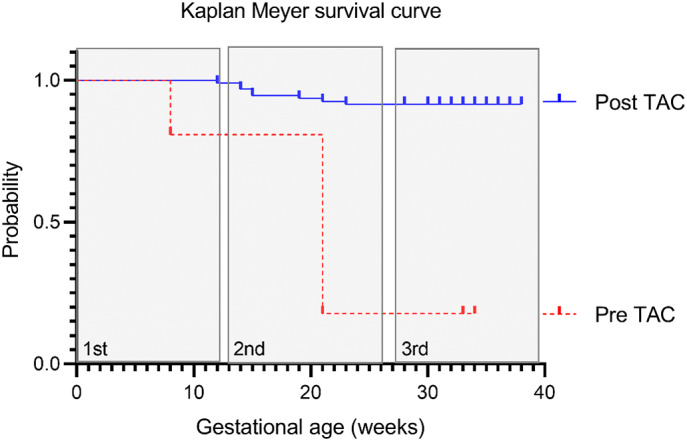
Kaplan–Meier survival curve. A direct comparison of pregnancy outcomes before and after the transabdominal cerclage (TAC) procedure within the same patients (*n* = 92). The Pre TAC curve includes data from 467 pregnancies before TAC with outcomes presented per trimester, as the exact gestational age (in weeks) of miscarriages was not available for the pregnancies before TAC. The data for Post TAC procedure indicates the outcomes of 118 pregnancies with the gestational age presented in weeks. Statistical analysis: χ^2^ test *P* < 0.001

The descriptive patient characteristics, indications for TAC as well as pregnancy outcomes before TAC (*n* = 94) are shown in Table 1. Before the index pregnancy, the median number of pregnancies per patient was 5 (range 1–9). Additional maternal pathology included body mass index of 30 kg/m^2^ or more (*n* = 12), chronic hypertension (*n* = 5, one with concomitant pregestational diabetes), previous myomectomies (*n* = 3, two with small fibroids still present), arcuate uterus (*n* = 2), and previous placental abruption (n = 2). Two cases living in deep rural areas presented additional challenges. Figure 2 depicts the improvement in pregnancy outcomes before and after TAC in the same patients. The χ^2^ analysis confirmed a significant (*P* < 0.001) improvement in the probability of survival post TAC.

**TABLE 1:** Descriptive details before transabdominal cerclage^a^

Characteristic	Values	
Number of cases	94 (100)	–
Age, years	–	31 ± 4
Gravidity	–	6 (2–10)
Parity	–	1 (0–4)
Previous failed vaginal cerclage	70 (74)	1 (1–3)
Indication for TAC		
Previous failed vaginal cerclage	18 (19)	–
Short vaginal cervix	13 (14)	–
Damaged vaginal cervix	13 (14)	–
Combination of above indications	50 (53)	–
Outcomes before TAC		
Number of pregnancies	467	5 (1–9)
All miscarriages	381	4 (0–9)
T1 miscarriages	89	1 (0–5)
T2 miscarriages	292	3 (0–7)
Number of viable pregnancies (≥27 weeks)	86	
Births ≥27 to 33^+6^ wk	32	1 (0–2)
Neonatal deaths ≥27 to 33^+6^ wk	8	–
Births >34 week	54	
Number of women with no viable pregnancies	41 (44)	–

Abbreviation: TAC, transabdominal cerclage.

^a^ Data are presented as number, number (percentage), mean ± standard deviation, or as median (range).

**FIGURE 3:**
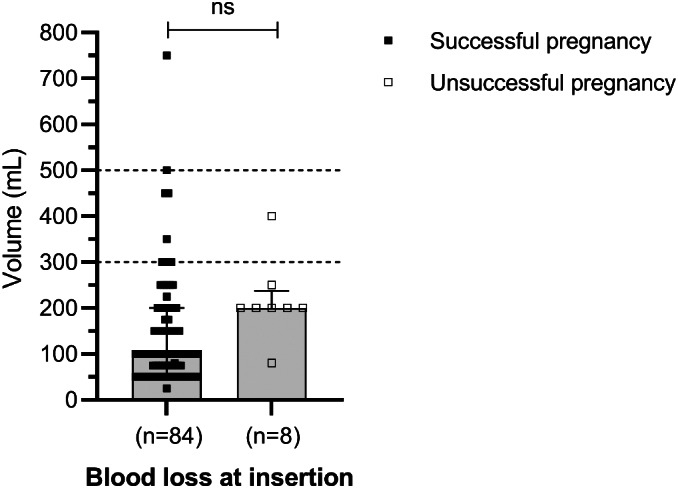
Blood loss during transabdominal cerclage insertion (*n* = 92). Statistical analysis: Welch *t*‐test with two‐tailed *P* value; ns, not significant

Descriptive details at the time of TAC surgery (*n* = 94) are shown in Table 2. The median blood loss at primary surgery was 100 ml (range 25–750 ml). Damage to venous branches adjacent to the supravaginal cervix that required hemostatic suturing, was noted in five cases (5/94). The highest intraoperative blood loss was 750 ml, where dilated venous complexes were present on both sides of the cervix, but the pregnancy involved progressed to 33 weeks. Delivery was for preterm, prelabor rupture of membranes and non‐reassuring cardiotocogram, but the baby was born with good Apgar scores. In three cases (3/94), blood was present in the vagina after the procedure. In two, the amount was minimal, but a 100 ml clot was present after an uncomplicated insertion in the other. All three cases had routine postoperative discharge and were delivered at term. Figure 3 depicts surgical blood loss in successful and unsuccessful pregnancies, with no association between blood loss and pregnancy outcome.

**TABLE 2:** Descriptive details at transabdominal cerclage surgery^a^

Characteristic	Value	
Number of cases	94 (100)	
Gestational age at TAC insertion (days)		92 ± 6 d (= 13^+1^ week)
Cervical length (vaginal ultrasound), mm		29.9 ± 9.29
Cervical beaking (vaginal ultrasound)	15 (16)	–
Blood loss at surgery, ml	–	100 (25–750)
Blood loss ≥300 ml	10 (11)	–
Blood loss ≥500 ml	2 (2)	–
Uncomplicated insertion	86 (91)	–

Abbreviation: TAC, transabdominal cerclage.

^a^ Data are presented as number (percentage), mean ± standard deviation, or as median (range).

Intraoperative membrane rupture occurred twice. Once, there was difficulty in replacing the gravid uterus through the Pfannenstiel incision of an obese abdomen with waning muscle relaxant. Amniotic fluid was clearly detectable vaginally after closure of the abdomen, but resealing was confirmed clinically and by ultrasound within 48 h. This patient delivered at term. The other intraoperative membrane rupture occurred at 14^+1^ weeks. As there was no re‐sealing within 48 h, the TAC was removed abdominally on day 2 and the pregnancy passed vaginally. In another case, membranes ruptured within 24 h after a TAC at 14^+3^ weeks. The fetus was passed vaginally before removal of the suture. One pressing case (gravidity 6, parity 0), underwent TAC beyond the “safe 12^+0^–13^+6^ weeks” gestation at 15^+4^ weeks. Membrane rupture occurred on postoperative day 2, the TAC was removed vaginally and the pregnancy was aborted. A second TAC procedure in her next pregnancy was successful. Lastly, a single intrauterine death was diagnosed on postoperative day 2 after insertion at 11^+5^ weeks. Three veins in the vicinity of the supravaginal cervix were ligated during insertion, with an operative blood loss of 80 ml.

**FIGURE 4:**
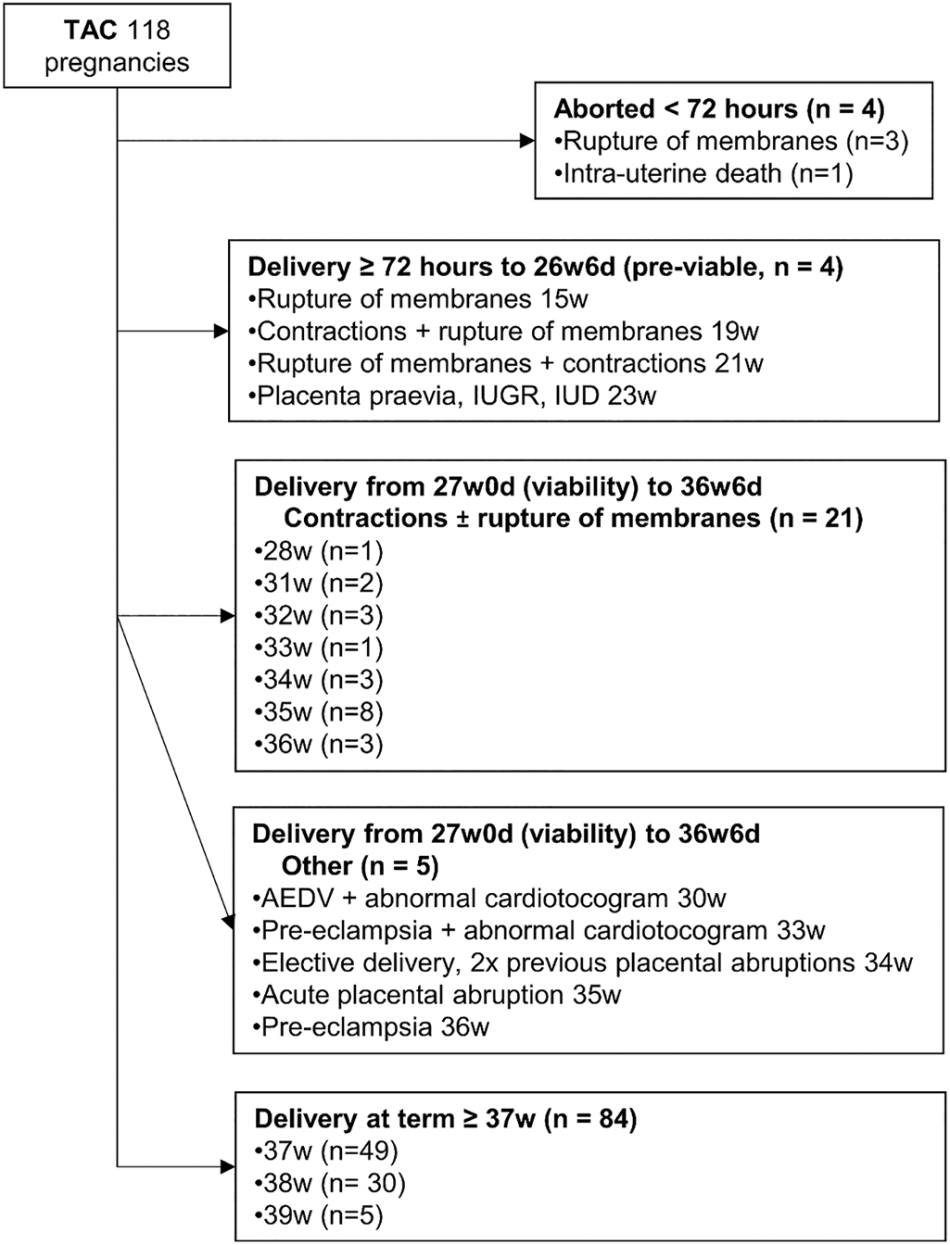
Chronologic gestational age and delivery indications post TAC. The data from 118 pregnancies in 92 patients are presented. AEDV, absent end diastolic velocimetry in umbilical artery; IUD, intrauterine death; IUGR, intrauterine growth restriction

The complications and delivery indications, from 72 h until the final delivery at term, are shown in three clinically relevant time periods in Figure 4. These include:

- Complications from 72 h to 26^+6^ weeks (pre‐viability, *n* = 4).
- Delivery from 27^+0^ weeks (viability) to 36^+6^ weeks (preterm period, *n* = 26). The earliest delivery here was at 28 weeks of pregnancy for fetal distress. That pregnancy was characterized by chronic hypertension, bilateral uterine artery notching on Doppler velocimetry at 22 weeks and poor fetal growth. Super‐imposed pre‐eclampsia was diagnosed at 24 weeks, and absent end‐diastolic umbilical artery flow velocimetry at 25 weeks. There had been no fetal growth in the 2 weeks preceding delivery. The baby weighed 600 g with meconium in the liquor and a pH of 7.2. Initial course was fair, with eventual discharge in a stable condition. The majority (21/26) of deliveries in this category were for contractions and/or rupture of membranes, with standard obstetric practice applied. Seven cases were delivered in the early preterm period (27^+0^–33^+6^ weeks), while 14 deliveries fell in the late preterm category. The other five cases were complicated by pre‐eclampsia, placental abruption, and placental dysfunction.
- Delivery at term 37 weeks or later (*n* = 84).

There was no evidence of an association between cervical length at TAC insertion and outcome. The cervical lengths for the successful outcome and no viable pregnancy groups were 30 ± 9 mm (mean ± standard deviation) (range 13–65 mm) and 27 ± 9 mm (range 17–40 mm), respectively. The bulk of deliveries occurred in the early term period. With the possibility of stitch erosion, either spontaneous or after contractions, the lower segment was carefully examined at each delivery. Stitch erosion occurred in seven cases, with five associated with contractions. None (0/7) were associated with maternal or fetal morbidity. For the singleton pregnancies that reached viability, the mean (± standard deviation) birth weight was 2747 ± 647 g. No 5‐min Apgar score was less than 7 and no early neonatal deaths occurred. Two sets of twins in second pregnancies after TAC were delivered at 31 and 34 weeks, respectively.

## 4 DISCUSSION

Transabdominal cerclage is a last resort intervention for recurrent mid‐trimester loss where vaginal cerclage has failed or is not possible.^6^, ^14^, ^16^ These highly selected women are estimated at 13% of cervical insufficiency cases,^17^ and are the most difficult in which to achieve a successful pregnancy. In our cohort, 41 (44%) of women had not achieved a viable pregnancy. Given this context, the 91% success rate after first insertion, is remarkable. This figure is similar to that of Sumners et al.,^15^ and a British study spanning 22 years, which included first‐trimester (59 cases) and preconception (62 cases) transabdominal cerclages.^10^ In the latter series, 93% of first‐trimester TACs were successful, with their more liberal success threshold of more than 24 weeks of pregnancy, 3 weeks earlier than in the current study. Seventy‐four per cent of their first‐trimester TAC pregnancies progressed beyond 34 weeks, compared with 86% in our study.

Sumners et al.,^15^ have described the intimidating risk of surgical hemorrhage from the paracervical veins as the most treacherous and complicated part of the procedure.^15^ When interpreting articles describing surgical blood loss, it is critical to note whether the procedure was performed before or during pregnancy, as pregnancy‐induced vascularization of the surgical field, decreased mobility, and the presence of a fetus, make the operation more difficult. In one series, 50% of the women whose procedures were performed in pregnancy experienced hemorrhage of more than 500 ml, whereas no surgical complications occurred in the preconception group.^10^ In our series, the median blood loss of 100 ml compares closely with 110 ml described in a recent systematic review and meta‐analysis.^13^ Although preconception TAC reduces intraoperative blood loss,^10^, ^13^ women in LMICs often present in pregnancy, which argues for the retention of this scarce skill. Other described intraoperative complications such as inadvertent cystotomy and bowel injury were not encountered in the index study.^15^, ^16^

Fetal death within 2 weeks of surgery has been quantified as <2% by Sumners et al.,^15^ which correlates with our single case. The systematic review and meta‐analysis by Marchand et al.^14^ found that both laparoscopic and open TAC are effective in prolonging gestational age, but neither is able to prevent all preterm deliveries. Dual pathology such as maternal disease or uteroplacental malperfusion is common.^10^ Contractions with/without rupture of membranes occurred in six cases before, and 21 (18%) cases after viability. Erosion of the 5‐mm wide, woven permanent tape through the cervical wall, either as a result of pressure or uterine contractions (or both), is well‐described, with a frequency of “rare” to 25%.^15^ Therefore, cases in which preterm labor occurs must be carefully monitored. The potential danger of stitch erosion also determines the timing of elective cesarean section. It is encouraging that none of the current stitch erosion cases were associated with maternal or fetal morbidity.

Most patients in this series presented with tragic obstetric histories and were helped by TAC with the advantage that it can be retained for subsequent pregnancies. The 24 (22 second, and 2 third) additional pregnancies (including two sets of twins) among 22 women who elected to retain the TAC after the first pregnancy, all of which were successful, add further hope to this often desperate, patient population. A long‐term study described up to five subsequent deliveries after TAC.^12^

The systematic review and meta‐analysis by Marchand et al.,^14^ noted that a major limitation of their review was a lack of studies. The present large “in context” study helps to address the data deficit. The usual limitations of a retrospective study were limited by the continuous quality control by the principal author over the entire period, with data of all cases familiar and available. The study duration, long follow up, and LMIC setting, are additional strengths.

## AUTHOR CONTRIBUTIONS

DRH conceived the study, wrote the protocol, collected the data, and wrote the manuscript. MvdV performed the data analysis and assisted with writing the manuscript.

## FUNDING INFORMATION

This research did not receive any specific grant from funding agencies in the public, commercial, or not‐for‐profit sectors.

## CONFLICT OF INTEREST

The authors have no conflicts of interest to declare.

## Data Availability

Research data are not shared.
